# Multicenter dose-escalation Phase I trial of mitomycin C pressurized intraperitoneal aerosolized chemotherapy in combination with systemic chemotherapy for appendiceal and colorectal peritoneal metastases: rationale and design

**DOI:** 10.1515/pp-2022-0116

**Published:** 2022-06-21

**Authors:** Mustafa Raoof, Kevin M. Sullivan, Paul H. Frankel, Marwan Fakih, Timothy W. Synold, Dean Lim, Yanghee Woo, Isaac Benjamin Paz, Yuman Fong, Rebecca Meera Thomas, Sue Chang, Melissa Eng, Raechelle Tinsley, Richard L. Whelan, Danielle Deperalta, Marc A. Reymond, Jeremy Jones, Amit Merchea, Thanh H. Dellinger

**Affiliations:** Department of Surgery, Division of Surgical Oncology, City of Hope National Medical Center, Duarte, CA, USA; Department of Computation and Quantitative Medicine, City of Hope National Medical Center, Duarte, CA, USA; Department of Medical Oncology and Therapeutics, City of Hope National Medical Center, Duarte, CA, USA; Analytical Pharmacology Core, City of Hope Comprehensive Cancer Center, Duarte, CA, USA; Department of Pathology, City of Hope National Medical Center, Duarte, CA, USA; Office of Clinical Research, City of Hope National Medical Center, Duarte, CA, USA; Department of Surgery, Northwell Health, Donald and Barbara Zucker School of Medicine, New Hyde Park, NY, USA; Department of Oncology (Medical), Mayo Clinic, Jacksonville, FL, USA; Department of Surgery, Mayo Clinic, Jacksonville, FL, USA; Department of Pathology, Northwell Health, New York, NY, USA; Department of Surgery, University of Tuebingen, Tubingen, Germany

**Keywords:** appendiceal cancer, colorectal cancer (CRC), mitomycin C (MMC), peritoneal metastasis (PM), Phase I study, pressurized intraperitoneal aerosolized chemotherapy (PIPAC)

## Abstract

**Objectives:**

Peritoneal metastasis (PM) from appendiceal cancer or colorectal cancer (CRC) has significant morbidity and limited survival. Pressurized intraperitoneal aerosolized chemotherapy (PIPAC) is a minimally invasive approach to treat PM. We aim to conduct a dose-escalation trial of mitomycin C (MMC)-PIPAC combined with systemic chemotherapy (FOLFIRI) in patients with PM from appendiceal cancer or CRC.

**Methods:**

This is a multicenter Phase I study of MMC-PIPAC (NCT04329494). Inclusion criteria include treatment with at least 4 months of first- or second-line systemic chemotherapy with ineligibility for cytoreductive surgery and hyperthermic intraperitoneal chemotherapy (CRS-HIPEC). Exclusion criteria are: progression on chemotherapy; extraperitoneal metastases; systemic chemotherapy intolerance; bowel obstruction; or poor performance status (ECOG>2). Escalating MMC-PIPAC doses (7–25 mg/m^2^) will be administered in combination with standard dose systemic FOLFIRI. Safety evaluation will be performed on 15 patients (dose escalation) and six expansion patients: 21 evaluable patients total.

**Results:**

The primary endpoints are recommended MMC dose and safety of MMC-PIPAC with FOLFIRI. Secondary endpoints are assessment of response (by peritoneal regression grade score; Response Evaluation Criteria in Solid Tumors [RECIST 1.1], and peritoneal carcinomatosis index), progression free survival, overall survival, technical failure rate, surgical complications, conversion to curative-intent CRS-HIPEC, patient-reported outcomes, and functional status. Longitudinal blood and tissue specimens will be collected for translational correlatives including pharmacokinetics, circulating biomarkers, immune profiling, and single-cell transcriptomics.

**Conclusions:**

This Phase I trial will establish the recommended dose of MMC-PIPAC in combination with FOLFIRI. Additionally, we expect to detect an early efficacy signal for further development of this therapeutic combination.

## Introduction

The presence of peritoneal metastases (PM) is a poor prognostic factor in patients with colorectal cancer (CRC) and appendiceal cancers (AC). Compared to patients with isolated liver or lung metastases, patients with PM have the worst overall survival with a median 14 months [1]. Quality of life is often impacted by complications of the disease including malignant bowel obstruction, small bowel dysfunction, ascites, or obstructive uropathy. These complications also limit the ability of patients to tolerate standard or clinical trial therapies.

A fraction of patients with peritoneum-limited disease benefits from curative-intent cytoreductive surgery (CRS). This subset of patients treated with perioperative systemic chemotherapy and optimal cytoreduction, demonstrate favorable overall survival (median 41 months) [2]. Over the last two decades, hyperthermic intraperitoneal chemotherapy (HIPEC) had become routine in many cancer centers. However, results from the PRODIGE 7 trial, a multicenter, randomized, open-label, Phase III trial of CRS-HIPEC vs. CRS alone for CRC patients with PM, showed no benefit of adding oxaliplatin HIPEC after complete CRS vs. CRS alone [2]. Further, despite the demonstrated safety and efficacy of CRS with or without HIPEC, most patients even with peritoneum-limited disease are not candidates for CRS due to an extensive disease burden. While first-line preoperative systemic therapy can downstage disease in 30–40% of patients [3], more effective treatments are needed to convert patients to resectability or provide durable disease control.

Pressurized intraperitoneal aerosol chemotherapy (PIPAC) is a novel approach for regional therapy being investigated for patients with CRC-PM [4]. It is performed minimally invasively via laparoscopy and is repeatable. Compared to other forms of intraperitoneal chemotherapy, pressurization, and aerosolization has been shown in preclinical models to distribute chemotherapy homogeneously and improve tissue penetration [5], [6], [7].

### Clinical experience with PIPAC in CRC and AC

Several retrospective and prospective studies have demonstrated the feasibility, safety, and tolerance of PIPAC in patients with PM from CRC [5, 8], [9], [10], [11], [12] and gastrointestinal cancer including AC [13], [14], [15]. Surgical complications are rare, with 3% intraoperative and postoperative complications reported in prospective studies. Overall, significant adverse events (grade >2) occur in 12–15% of procedures [8]. Most frequently cited side effects include abdominal pain, nausea, and fatigue.

Three dose-finding Phase I studies of PIPAC with oxaliplatin have been published (Table 1). The PIPOX trial (NCT03294252) [16, 17] demonstrated a maximum tolerated dose (MTD) of 90 mg/m^2^ for PIPAC with oxaliplatin in 10 patients with gastrointestinal (GI) cancers (5 CRC, 3 gastric, 2 small bowel cancer). No dose-limiting toxicities (DLTs) were observed at 90 mg/m^2^; and two DLTs at 140 mg/m^2^. Oxaliplatin concentrations were three- to four-fold higher in tissues that were in contact with aerosol than in muscle without contact. Systemic chemotherapy (fluorouracil [5-FU] and leucovorin) was allowed between PIPAC sessions. The histologic peritoneal regression grading score (PRGS) [18] demonstrated no patients with grade 4 or no response (no regression and tumor cells visible at lowest magnification), 5 patients with grade 3 or minor response (tumor cells predominant over fibrosis), 2 patients with grade 2 or major response (regressive changes such as necrosis, fibrosis, or acellular mucin predominant over tumor cells), and 2 patients with grade 1 or complete response (no tumor cells, only abundant fibrosis, acellular mucin, or necrosis). Patients with PRGS 1 underwent complete CRS and HIPEC. Some patients experienced a major histological response allowing a secondary complete resection. The study is open for Phase II enrollment.

**Table 1:** Dose finding Phase I studies of PIPAC.

Trial	Oxaliplatin dose	Patients	Toxicities	Efficacy
PIPOX [16, 17]	90 mg/m^2^ MTD	10	No DLT at MTD	All patients at least minor response by PGRS
PIPAC-OX [11]	120 mg/m^2^ RP2D	16	No AEs at RP2D	PCI decrease from 15 to 12, and PRGS from 2.5 to 2.0.
Robella et al. [12]	135 mg/m^2^ RP2D	6	Grade 1–2 abdominal pain	Not assessed

MTD, maximum tolerated dose; DLT, dose limiting toxicities; PRGS, peritoneal regression grading score; RP2D, recommended Phase II dose; AE, adverse events; PCI, peritoneal carcinomatosis index.

In contrast, the Phase I PIPAC-OX trial demonstrated a recommended Phase II dose (RP2D) of 120 mg/m^2^ [11] based on 16 evaluable patients (5 CRC, 8 gastric, 1 appendiceal cancer). No PIPAC oxaliplatin–related adverse events (AEs) were reported at the RP2D level. Half the study cohort underwent two PIPAC procedures, with improvement of median peritoneal carcinomatosis index (PCI) [19] from 15 to 12, and PRGS from 2.5 to 2.0. This study identified acute pancreatitis as a previously unreported AE for in relation to PIPAC with oxaliplatin.

Another dose-finding Phase I study of PIPAC with oxaliplatin recently reported an RP2D of 135 mg/m^2^, however this dose was based on a single dose PIPAC, rather than multiple PIPAC cycles [12]. No systemic chemotherapy was used in this study.

Oncologic efficacy of PIPAC with oxaliplatin has been reported as third-line treatment for CRC patients, with a clinical response (as assessed by radiographic, pathologic or PCI improvement) of 71–86% and a median survival 15.7 months [20]. A CRC-PIPAC single-arm Phase II trial treated 20 patients with isolated, unresectable CRC PM in any line of palliative treatment with PIPAC-oxaliplatin at 92 mg/m^2^ every 6 weeks. Response rates were 0% (radiological), 50% (biochemical), 56% (pathological), and 56% (ascites). Median progression-free and overall survival was 3.5 months (interquartile range [IQR] 2.5–5.7) and 8.0 months (IQR 6.3–12.6), respectively. Major treatment-related AEs occurred in three of 20 (15%) patients after five of 59 (8%) procedures (abdominal pain, intraperitoneal hemorrhage, iatrogenic pneumothorax, and transient liver toxicity), including one possibly treatment-related death (sepsis of unknown origin) [21]. The ongoing CRC-PIPAC-II Dutch Phase II study is evaluating first-line electrostatic PIPAC (e-PIPAC) with oxaliplatin and systemic chemotherapy in CRC PM [22].

A recent international retrospective cohort study of patients with CRC-PM compiled 256 non-selected patients and 606 patients who underwent PIPAC from 17 eligible centers [23]. Over half (55.4%) received PIPAC with oxaliplatin as third-line treatment. A total of 104/256 (41%) patients had ≥3 PIPACs with 16.4% with partial remission, 20.3% stable by RECIST; mean PRGS: 2.6 ± 0.8 (baseline) vs. 2.1 ± 0.9 at PIPAC3 (p=0.001); median PCI was 21 (IQR15-29) at baseline and 20 (IQR12-27) at PIPAC3 (p=0.02); median OS was 11 months (IQR7.1–17.7) from 1st PIPAC [23].

Another retrospective study of 74 PIPAC–oxaliplatin procedures in 24 patients with CRC-PM demonstrated 21% had complete response and a median survival of 37.6 months from the time of PM diagnosis, or 20.5 months from the first PIPAC, with two cases of severe postoperative complications [24]. Among appendiceal cancer patients, a registry study of 5 patients with PM from appendiceal cancer were treated with PIPAC–oxaliplatin, with overall disease control rate (stable disease or clinical response) at second PIPAC of 67% [25].

### Systemic chemotherapy in combination with PIPAC

PIPAC was administered as monotherapy in most of the above studies, while in practice; many patients receive PIPAC in combination with systemic chemotherapy [26]. A systematic review of 12 studies (386 patients) reported that 44% patients received PIPAC with multimodal therapy [27]. Both the PIPAC-OX and PIPOX trials allowed sensitizing dose of 5-FU/leucovorin, though no other systemic chemotherapy was specified. As tolerance and cumulative toxicity likely differs for PIPAC with multimodal therapy, further studies are required to determine the optimal dose of PIPAC drugs in combination therapy.

### Rationale for use of mitomycin C in PIPAC

Mitomycin C (MMC) has been demonstrated as an active drug in combination with HIPEC in patients with appendiceal cancer and CRC, usually at a dose of 25–35 mg/m^2^ [28], [29], [30], [31]. Mitomycin C binds with DNA, resulting in inhibition of DNA synthesis. In higher doses, MMC also suppresses cellular RNA and protein synthesis. Mitomycin C is an excellent choice for intraperitoneal (IP) delivery because it is non cell cycle-specific, and thus has direct cytotoxic effect even after a short exposure. It has a large molecular weight that allows high exposure to peritoneal cavity compared to systemic circulation when delivered intraperitoneally. It is water soluble and is rapidly cleared from the systemic circulation. At the most-commonly administered dose (35 mg/m^2^), 28% of patients have grade 3/4 leukopenia [30]. A dose of 25 mg/m^2^ for HIPEC-MMC results in a 10% incidence of grade 3/4 leukopenia [30].

Use of MMC in PIPAC has not been extensively evaluated. Alyami et al. reported MMC as PIPAC monotherapy at a dose of 1.5 mg/m^2^ in 50 mL NaCl 0.9% in six patients with CRC PM due to allergy to platinum chemotherapy [32]. In an international survey of 62 PIPAC centers in 2018, six centers (10%) used a MMC protocol, at a 1.5 mg/m^2^ dose, if a formal contraindication to oxaliplatin existed [33]. Similarly, anecdotal reports include use of MMC for CRC patients who were unable to proceed to oxaliplatin due to hypersensitivity and allergic reactions (SP Somashekar, personal communication). The dose of MMC used in these patients was based on a preclinical animal study that used 14 mg MMC diluted in 50 mL NaCl 0.9%, which is approximately the equivalent of 7–8 mg/m^2^ [34]. In this setting, PIPAC with MMC as monotherapy was well-tolerated and resulted in clinical responses (email correspondence, unpublished). PIPAC with MMC as multimodal therapy has not been investigated. Further, there are currently no evidence based optimal MMC dose reports in humans for PIPAC. Consideration for using MMC in PIPAC for patients with appendiceal cancers and CRC thus requires a dose-finding study. The primary objective of the proposed Phase I trial is to identify the MTD of PIPAC with MMC and evaluate safety in patients with PM due to appendiceal cancer or CRC when delivered in combination with systemic chemotherapy.

## Materials and methods

This study will be conducted with the principles set forth in The Belmont Report: Ethical Principles and Guidelines for the Protection of Human Subjects or Research and the Declaration of Helsinki. This study was approved by the City of Hope Institutional Review Board (IRB) (ID #19184) and is being conducted under an open US multicenter Phase I trial (NCT04329494) investigating PIPAC in patients with ovarian, uterine, gastric, appendiceal cancer, or CRC with PM who have failed at least one previous standard chemotherapeutic treatment.

### Inclusion and exclusion criteria

Inclusion criteria for patients in the PIPAC-MMC arm include patients with appendiceal cancer or CRC with PM who are ≥18 years of age, Eastern Cooperative Oncology Group (ECOG) performance status (PS) ≤ 2, and no contraindications to laparoscopy. In addition, patients must have received at least 4 months (8 cycles) of first- or second-line standard-of-care systemic chemotherapy such as leucovorin, 5-FU, and oxaliplatin (FOLFOX), leucovorin, 5-FU, and irinotecan (FOLFIRI), or leucovorin, 5-FU, oxaliplatin, and irinotecan (FOLFOXIRI), with or without a biologic agent. If irinotecan-based chemotherapy was used, the patients should not have progression on irinotecan-based chemotherapy. Exclusion criteria include progression on both first- and second-line systemic therapy, progression on irinotecan-based chemotherapy, hematologic toxicities requiring significant dose reductions while on systemic therapy, bowel obstruction, life expectancy <6 months, simultaneous tumor debulking with gastrointestinal resection, severe medical comorbidities or laboratory abnormalities, and major systemic infection (Table 2). If patients have had dose reductions on any prior non-irinotecan-based chemotherapy for hematologic toxicities, the investigators will require evidence of tolerability of FOLFIRI for 2–4 cycles.

**Table 2:** Inclusion and exclusion criteria.

Inclusion	Exclusion
Documented informed consent	Extraperitoneal metastatic disease
Age ≥ 18 years	Progression on both first- and second-line systemic chemotherapy; progression on irinotecan-based chemotherapy
Histologically confirmed appendiceal or colorectal carcinoma	Bowel obstruction requiring nasogastric or gastrostomy tube
No contraindications for laparoscopy	Life expectancy of less than 6 months
Visible peritoneal disease on cross sectional imaging or on laparoscopy	Ascites due to liver cirrhosis or portal vein thrombosis
At least 4 months of standard of care systemic chemotherapy (e.g. FOLFOX, FOLFIRI, FOLFOXIRI). If irinotecan-based chemotherapy was used the patients should not have progression on irinotecan-based chemotherapy	Simultaneous tumor-debulking with gastrointestinal resection
	Uncontrolled current cardiac or renal comorbidity, myelosuppression, or hepatic impairment
	Exclusive total parenteral nutrition

### PIPAC procedure

**Figure 1:**
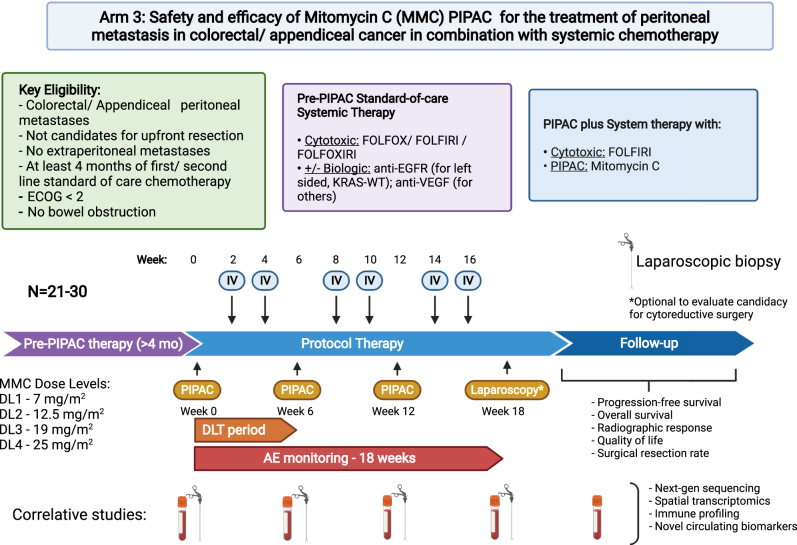
Study schema. Schema and overview of the Phase I trial of mitomycin C PIPAC in combination with systemic chemotherapy for colorectal and appendiceal cancers. Three PIPAC procedures are planned 6 weeks apart with two doses of IV systemic chemotherapy (FOLFIRI) every two weeks between PIPAC procedures. The starting dose of mitomycin C will be 7 mg/m^2^ and escalate to 12.5, 19, and finally 25 mg/m^2^. Following the procedures, progression-free survival, overall survival, radiographic response, quality of life, and treatment with cytoreductive surgery will be monitored. Samples will be taken at each PIPAC procedure for correlative transcriptional and genomic studies. PIPAC, pressurized intraperitoneal aerosolized chemotherapy; FOLFOX, folinic acid (leucovorin), fluorouracil (5-FU), oxaliplatin; FOLFIRI, folinic acid (leucovorin), fluorouracil (5-FU), irinotecan; MMC, mitomycin C; FOLFOXIRI, folinic acid (leucovorin), fluorouracil (5-FU), oxaliplatin, irinotecan; AE, adverse event; DLT, dose limiting toxicity; ECOG, Eastern Cooperative Oncology Group; IV, intravenous; DL, dose level

A detailed description of establishing a PIPAC program and technical considerations including occupational safety has been previously described [35]. Each subject is expected to receive up to 3 PIPAC treatments approximately 6 weeks apart (weeks 0, 6, 12) (Figure 1). Patients will receive concurrent systemic chemotherapy (FOLFIRI) every 2 weeks except for when receiving PIPAC therapy (weeks 2, 4, 8, 10, 14, 16). During laparoscopy, a 12 mm balloon port and a 5 mm balloon port will be placed to maintain adequate seal and prevent leakage of the pneumoperitoneum. Ascites will be removed, PCI determined, and peritoneal biopsies will be taken, but no lysis of adhesions or tumor resection will be performed due to the risk of increased morbidity. MMC dissolved in 150 mL of 0.9% NaCl will be used in a dose finding trial design starting at 7 mg/m^2^ and escalating to 25 mg/m^2^ based on observed toxicity data (Table 3). The dose levels will include 3 evaluable subjects at 7 mg/m^2^, followed by 3 at 12.5 mg/m^2^, 3 at 19 mg/m^2^, and 3+3 at 25 mg/m^2^ (Total 15 during dose escalation). An additional 6 patients will be accrued during expansion for a total of 21 evaluable patients (Supplementary Table S1), The drug will be administered using a high-pressure injector and a nebulizer (Capnopen; Capnomed GmbH, Germany) at a maximum of 200 psi and 30 mL/min. After aerosolization, the drug will be allowed to precipitate over a duration of 30 min at room temperature. The remaining aerosol will be evacuated.

**Table 3:** Rules for dose finding to identify MTD of MMC.

Dose level	Dose	Expected N (evaluable)	Comments
DL 1	7 mg/m^2^	3	Assuming no DLTs
DL 2	12.5 mg/m^2^	3	Assuming no DLTs
DL 3	19 mg/m^2^	3	Assuming no DLTs
DL 4	25 mg/m^2^	3+3	Assuming at most 1 DLT

MTD, maximum tolerated dose; DL, dose level; DLT, dose limiting toxicity.

### Rationale for dose levels

Clinical experience with MMC is derived from both systemic (IV) and HIPEC administration. The established IV MMC dose is 10 mg/m^2^ [36], while HIPEC MMC doses range from 10 mg/m^2^ to 35 mg/m^2^ [37]. (Table 4). Few PIPAC centers have administered PIPAC-MMC at 1.5 mg/m^2^ for platinum-allergic patients, which represent less than 10% of the typical systemic or HIPEC MMC dose [32, 33]. As anecdotal experience with a dose level of 7 mg/m^2^ (based on preclinical dose of 14mg/50 mL) exists from a large Indian PIPAC center (SP Somashekar, personal communication), with good tolerance in at least four PIPAC patients, we will begin dose escalation at a starting dose level of 7 mg/m^2^. As systemic exposure of MMC after HIPEC 40 mg/m^2^ is approximately equivalent to 15–20 mg/m^2^, we desired to achieve a maximum dose level of no greater than currently used maximum HIPEC doses. Thus, a maximum dose level of 25 mg/m^2^ is planned for this dose escalation study, with a total of four dose levels (7, 12.5, 19, and 25 mg/m^2^).

**Table 4:** Summary of chemotherapeutic agents and their Phase I studies of their use in PIPAC for CRC patients with PM.

	Oxaliplatin	Mitomycin C	Irinotecan^a^
IV dose, mg/m^2^	85	10	120–185
HIPEC dose, mg/m^2^	200–460	10-35 [30]	200
PIPAC dose, mg/m^2^	120 [11]	1.5–7 [32, 34]	20 [32, 33]
	90 [17]		
	135 [12]		
	92^b^		
Dose-escalation Phase I Study	Yes	No	No
RP2D	90–135 mg/m^2^	n/a	n/a
Efficacy	16.4% partial response	n/a	n/a
	20.3% stable response (RECIST)		
Grade ≥ 3 Toxicity	Pancreatitis, neutropenia, allergic reaction, pain, nausea, peripheral neuropathy	n/a	n/a

^a^In 2018 international survey, only one center used Irinotecan PIPAC [33]. ^b^arbitrary dose. RP2D, recommended Phase II dose; IV, intravenous; HIPEC, hyperthermic intraperitoneal chemotherapy; PIPAC, pressurized intraperitoneal aerosolized chemotherapy; RECIST 1.1, Response Evaluation Criteria in Solid Tumors.

### Statistical analysis

The safety evaluation is expected to include 15 patients during the dose escalation and an additional 6 expansion patients for a total of 21 evaluable patients. The safety evaluation period is 6 weeks. Dose escalation will be performed using standard 3+3 rules [38]. If 0 out of 3 patients experience a dose-limiting toxicity (DLT), 3 patients will be entered at the next dose level. If 2 or more of 3 patients experience a DLT, then escalation will be stopped, and the maximum dose is declared. If 1 out of 3 patients experience a DLT, 3 additional patients will be entered, and if another patient experiences toxicity, then escalation will be stopped and the maximally administered dose is declared.

## Results

### Feasibility of the study

Patients will be recruited at multiple sites across the US including City of Hope National Medical Center, Northwell Health, and the Mayo Clinic. Investigators at all sites have undergone PIPAC certification by International Society for Study of Pleura and Peritoneum (ISSPP). All sites have been actively accruing CRC patients for PIPAC oxaliplatin administration since 2020 and will implement the MMC PIPAC protocol as a third arm of US PIPAC trial (NCT04329494).

### Primary endpoints

The primary endpoints of this study are the RP2D and safety with determination of DLTs. The endpoint of RP2D will be determined using a traditional 3+3 dose escalation to determine the maximum tolerated dose. Adverse events will be graded per Common Terminology Criteria for Adverse Events (CTCAE) criteria. DLTs will include grade 3 or 4 nonhematologic toxicities, excluding grade 3 nausea, vomiting, abdominal pain, diarrhea, fatigue, or laboratory abnormalities that return to or are correctable to grade 2, and grade 3 peripheral neuropathy. Other DLTs including Clavien-Dindo [39] grade IIIB or higher surgical complications, grade 4 thrombocytopenia or neutropenia, or delay of over 21 days to second cycle of PIPAC due to a PIPAC-related adverse event.

### Secondary endpoints

Secondary endpoints for efficacy will be determined by evaluating response via three separate criteria. First, for PM evident on cross sectional imaging, (RECIST) by CT scan will be measured. Efficacy will be defined as percentage of evaluable patients who achieve complete or partial response, or stable disease. Second, PRGS will be evaluated at each cycle, with both preoperative and postoperative peritoneal samples. Efficacy will be defined as percentage of patients who have achieved a decrease in PRGS over successive biopsies. Third, at each laparoscopy, (PCI) will be measured and changes evaluated over time. Efficacy will be defined as percentage of patients who have achieved complete response, partial response, or stable disease. Surgical complications by Clavien-Dindo classification will be evaluated at 4 weeks after each PIPAC. We will monitor the progression-free survival (PFS) at 1 year and rate at which CRS is performed. Finally, functional status as determined by daily steps tracked with Vivofit 4 (Garmin) wristband pedometer and patient-reported quality of life symptoms will be measured by the EQ-5D-5L and MD Anderson Symptom Inventory before treatment and at 6, 12, and 18 weeks. Technical failures of PIPAC will also be recorded. Technical failure is defined as inability to gain access to the abdomen, malfunction of equipment, too much adhesive disease to allow dispersion of the treatment, or >5 L ascites. The technical failure rate is calculated as technical failure percentage of all procedures.

## Discussion

There are several novel aspects to the proposed study that merit discussion:

1. PIPAC with MMC has not been systematically investigated in patients with CRC or appendiceal cancer PM. This is the first multicenter study to test the safety and feasibility of PIPAC with MMC. We expect to identify the RP2D at the completion of the study.
2. While PIPAC trials for unresectable CRC with PM have focused on oxaliplatin therapy, efficacy has been limited. This is because most colorectal and appendiceal cancer patients included in PIPAC trials are already refractory to systemic oxaliplatin. PIPAC with MMC minimizes the problem of cross-resistance and is likely to retain efficacy in oxaliplatin–refractory patients.
3. PIPAC with MMC will provide an alternative option for patients with severe platinum allergies who are not candidates for PIPAC–oxaliplatin [40].
4. The majority of PIPAC studies have included patients who have progressed on multiple lines of systemic chemotherapy. At advanced stages of chemotherapy refractory disease, the peritoneal tumors can be bulky, limiting efficacy of regional therapy such as PIPAC. This study is innovative because it moves PIPAC to an earlier time point prior to patients becoming refractory to irinotecan-based systemic chemotherapy.
5. The proposed trial will evaluate the safety of PIPAC with MMC in combination with systemic chemotherapy (FOLFIRI). Many patients with unresectable CRC or appendiceal cancer PM likely have micro-metastatic disease. While PIPAC may be able to control PM, prior studies have shown minimal systemic absorption. As such regional delivery of PIPAC-based therapy is unable to address micro-metastatic systemic disease. The trial proposes a pragmatic framework to comprehensively treat PM in these patients.

## Supplementary Material

Supplementary Material Details
