# Supplementary material for: Multicenter dose-escalation Phase I trial of mitomycin C pressurized intraperitoneal aerosolized chemotherapy in combination with systemic chemotherapy for appendiceal and colorectal peritoneal metastases: rationale and design
Source: Pleura Peritoneum. 2022 Jun 21;7(4):169–77. doi: 10.1515/pp-2022-0116 (PMC9742457; doi:10.1515/pp-2022-0116)
Supplement: Supplementary file 1 — Supplementary Material Details [file j_pp-2022-0116_suppl.docx]

**Supplementary Table 1:** Dose escalation design

| **Number of Patients with DLT at a Given Dose Level** | **Escalation Decision Rule** |
| --- | --- |
| 0 out of 3 | Enter 3 patients at the next dose level. |
| ≥ 2 | Dose escalation will be stopped. This dose level will be declared the maximally administered dose (highest dose administered). Three (3) additional patients will be entered at the next lowest dose level if only 3 patients were treated previously at that dose. |
| 1 out of 3 | Enter at least 3 more patients at this dose level.   - If 0 of these 3 patients experience DLT, proceed to the next dose level. - If 1 or more of this group suffer DLT, then dose escalation is stopped, and this dose is declared the maximally administered dose. Three (3) additional patients will be entered at the next lowest dose level if only 3 patients were treated previously at that dose. |
| ≤ 1 out of 6 at highest dose level below the maximally administered dose | This is generally the RP2D. At least 6 patients must be entered at the RP2D. |

Multiple simulation studies examining the operating characteristics of the traditional 3+3 rules, have demonstrated that on average, the standard 3+3 rules tend to identify a maximum tolerated dose (MTD) with a DLT rate between 10-25%. Empirically, the dose limiting toxicity (DLT) rate at the recommended phase II dose (RP2D) will either be 0% (0/6) or 16.7% (1/6), with safety confirmed during the expansion cohort to increase the experience at the RP2D to 12 patients. During the expansion cohort if the DLT rate observed equals 25% or higher, accrual will hold pending review by the DSMB, Institutional Review Board (IRB), sponsor-Investigator, and Food and Drug Administration (FDA).
